# A Holistic In Silico and In Vivo Approach to Exploring the Antidiabetic, Antioxidant, and Hepatoprotective Properties of Rose of Sharon

**DOI:** 10.3390/life14060686

**Published:** 2024-05-27

**Authors:** Sedef Ziyanok-Demirtas

**Affiliations:** Department of Biology, Faculty of Science and Arts, Bursa Uludag University, Bursa 16059, Turkey; sziyanok@uludag.edu.tr

**Keywords:** *Hibiscus syriacus*, antioxidant, hepatoprotectivity, hyperglycemia, molecular docking, oxidative stress, type 1 diabetes

## Abstract

Diabetes mellitus (DM) is a significant global health burden that necessitates the exploration of effective and accessible therapeutic options. Phytotherapy has played a vital role in healthcare, with plant extracts being integral to traditional medicinal practices. The therapeutic potential of *Hibiscus syriacus* (Rose of Sharon), a plant with a rich ethnobotanical history, in the management of DM and its associated complications was investigated. In this study, the therapeutic potential of *Hibiscus syriacus* L. extract (HSE) against DM in streptozotocin (STZ)-induced diabetic rats was assessed, focusing on its effects on glucose regulation, antioxidative defense, and liver protection. The administration of the HSE extract substantially reduced hyperglycemia and increased insulin production, with concurrent improvements in body weight and hydration. The enhanced activity of antioxidant enzymes, such as superoxide dismutase (SOD) and glutathione peroxidase (GSH-Px), suggests reduced oxidative stress, which is further supported by molecular docking results with the 3GTV superoxide dismutase enzyme, showing a binding energy of −6.3 kcal/mol. A decrease in MDA levels also indicates a reduction in oxidative stress. Notably, HSE treatment led to decreased aspartate aminotransferase (AST) and alanine aminotransferase (ALT) levels and improved lipid profiles, indicating its hepatoprotective and lipid-modifying benefits. These findings support the inclusion of HSE as an adjunctive therapy in DM management strategies. This study promotes the consideration of *Hibiscus syriacus* L. therapeutic properties in global health contexts.

## 1. Introduction

Throughout history, phytotherapy has served as the foundation for therapeutic remedies, with numerous efforts being made to harness herbal treatments in battle against various diseases. This practice can be characterized as the application of plant-based remedies for the prophylaxis and therapeutic management of ailments. Any part of the plant, be it the leaf, flower, seed, root, or bark, utilized for medicinal interventions is termed a “phytotherapeutic agent” or “medicinal plant extract” [1,2].

Plant extracts have been a cornerstone of traditional medicine for millennia, offering a plethora of therapeutic benefits derived from the natural abundance of botanical diversity. Civilizations across the world, from ancient Chinese and Ayurvedic traditions to Greek and Roman practices, have long harnessed the power of plants to alleviate ailments, restore health, and promote overall well-being. With the advent of modern pharmacology, the exploration of the potential of plant extracts has only intensified, marrying traditional knowledge with scientific rigor. As the global community faces myriad health challenges, from chronic diseases to antibiotic-resistant infections, the use of plant extracts remains undiminished. Their complex phytochemical compositions, encompassing flavonoids, alkaloids, terpenoids, and essential oils, to name a few, present vast opportunities for drug discovery and the development of novel therapeutic agents. Moreover, in an era increasingly marked by a return to natural and holistic remedies, plant extracts are heralded not only for their potential efficacy but also for their perceived safety and harmony with the human body [3,4].

Diabetes mellitus (DM) is a chronic metabolic disease characterized by high blood sugar levels resulting from a deficiency in the pancreas’ insulin production or a decreased sensitivity of cells to insulin. This condition is often called “diabetes” and can lead to serious complications or even death if left untreated [5]. Diabetes is the sixth leading cause of death in the world due to its associated complications. The increasing number of diabetic patients imposes a significant financial burden on the healthcare system [6]. There is an urgent need for cost-effective and effective treatment options to manage diabetes, which requires not only medication but also lifestyle changes, such as exercise, diet, and regular check-ups. Approximately 43% of diabetic patients worldwide, including Turkey, cannot reach their treatment goals. This low compliance can be attributed to problems with the system, limitations of existing treatments, and patients’ reluctance to follow treatment. The chronic and progressively worsening nature of diabetes often results in feelings of hopelessness and fatigue. The current dearth of efficacious treatments for diabetes has necessitated the pursuit of herbal remedies by those afflicted with the disease in search of an alternative and dependable therapeutic modality [7,8,9].

Recent research has focused on plants with antidiabetic effects, and Hibiscus species have shown promising results in regulating blood glucose levels and lipid balance. *Hibiscus syriacus*, also known as Rose of Sharon, is a flowering plant native to Southern Europe, Asia, and Africa. Its medicinal properties have been recognized for centuries, and various active components in the plant are attributed to its broad range of health benefits [10]. *H. syriacus* has beautiful flowers and a long flowering period; it is often grown in gardens or as a hedge. Because of its availability and convenience, it is widely used in the Far East as a food source in times of food shortages. For example, steamed leaves are used as food, and the leaves are added to rice cakes and tea [11]. In traditional medicine of the Far Eastern countries, the dried root and stem bark are used as an antidote, as a spring tonic, and to reduce fever [12]. *H. syriacus* is a medicinal plant used as an antipyretic and anthelmintic. Its anti-inflammatory activity, ability to lower cholesterol effects/serum cholesterol levels, ability to prevent heart attacks, role as a mild laxative, ability to address urinary tract problems, cleansing and detoxification of skin and vascular health, and antifungal agent effects have also been reported [13]. The results from the Park et al. [14] study strongly suggest the possibility of excellent pharmacological activity and nutritional advantages not only in the petals but also in every part of the *H. syriacus* plant. Although it has many uses, most of the biological functions of *H. syriacus* L. are not clearly clarified.

Molecular docking techniques, which are based on accurately predicting the interaction energies for various potential binding modes and conformations, enable the elucidation of molecular interactions within biological systems [15]. This approach is crucial for predicting how plant-derived compounds interact with biological targets and for elucidating biological pathways.

Our previous study has demonstrated the antioxidant, hypoglycemic, hypolipidemic, and hepatoprotective properties of tea derived from *Hibiscus trionum* in diabetes mellitus-induced rats. This study indicated that *H. trionum* possesses remarkable in vivo antioxidant potential and contains a significant quantity of phenolics, such as kaempferol and quercetin [16]. Building on this, the current investigation was undertaken to evaluate the effects of the methanolic extract of *Hibiscus syriacus* on glucose metabolism, lipid profile, antioxidant status, and hepatoprotection in diabetic rats.

## 2. Materials and Methods

### 2.1. Plant Materials and Extraction Methods

Aerial parts of *H. syriacus* were collected from Kahramanmaras, Turkey, and were identified by Professor. Dr. Ahmet Ilcim. The diseased portions were excised. To decontaminate the plant samples, they were soaked in 30% ethanol for 10 min. The leaf samples were then washed sequentially with tap water and distilled water. The samples were then air-dried in the shade. Once fully dried, the samples were finely ground in a blender (Arcelik, Bursa, Turkey). From the ground samples, 5 g were weighed and combined with 50 mL of methanol. This process was repeated thrice to ensure consistency. The mixtures were then incubated in an oven at 40 °C for 24 h. The resultant solutions were centrifuged at 4500 rpm. The supernatant was collected, separated using an organic solvent evaporator (Bursa, Turkey), and stored at +4 °C for preservation.

### 2.2. Chemical Composition Analysis of Hibiscus syriacus

Freshly prepared *Hibiscus syriacus* extract was administered to animals and analyzed twice with an HPLC-DAD (1200) system to identify phenolic compounds following the method described by Aybastier et al. [17]. The analysis utilized a mobile phase gradient comprising 1% formic acid in water (component A) and acetonitrile (component B). The gradient started with 10% B, increased to 13% B over the first 10 min, rose to 41.5% B at 20 min, reached 70% B at 25 min, and then returned to 10% B between 35 and 38 min. An XBridge C18 column ((4.6 × 250 mm, 3.5 μm); Waters, Ireland) was used to separate phenolic compounds. The injection volume of each sample was 10 μL, with a consistent flow rate of 0.5 mL/min. The peaks were identified by matching the UV spectra and retention times with the established standards for phenolic compounds. Gallic acid and catechin standards used in the peak evaluations were commercially obtained from Sigma (MO, USA).

### 2.3. Animals

In the present study, we used 36 adult male Wistar rats, each with a body weight ranging from to 350–400 g. The rats were maintained in accordance with standard laboratory protocols and were provided with unrestricted access to food and water. The experimental procedures were approved by the Committee for the Care and Use of Laboratory Animals at Bursa Uludag University, Bursa, Turkey, under approval number 2018-06/07. The housing conditions were maintained at a constant room temperature of 25 ± 2 °C with a relative humidity of 55 ± 5%, and a 12 h light–dark cycle was observed. Throughout the study, all efforts were made to uphold the highest both national and international ethical standards and ensure the well-being of animals. Within each experimental group, control animals (*n* = 9) and diabetic animals (*n* = 9) were housed separately in their own individual cages. *Hibiscus syriacus* (HSE) was administered by gavage every early morning to both diabetic and healthy animals.

### 2.4. Diabetes Induction

**Figure 1:**
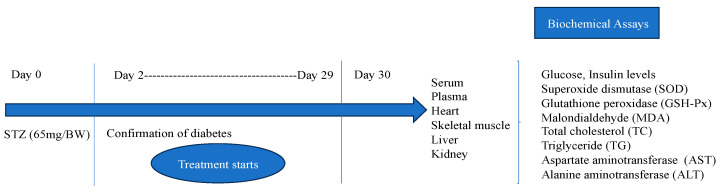
Experimental design.

Type 1 diabetes was induced by administering a single intraperitoneal injection of streptozotocin (STZ) at a dose of 65 mg/kg (pH 4.5) to rats that fasted overnight. The STZ was sourced from Sigma-Aldrich, St. Louis, MO, USA. The control group rats (C) received only citrate buffer. Blood glucose levels were measured two days after the streptozotocin injection. In our study, rats with blood glucose levels of 200 mg/dL or higher were considered diabetic and were included in subsequent experiments. The experimental design is shown in Figure 1.

### 2.5. Grouping of Animals

Animals were randomized and divided into four groups as the healthy rats (control group, Untreated) “C”, the healthy rats administered with *Hibiscus syriacus* extract “C + HSE”, the diabetic rats (positive control) “D”, and the diabetic rats administered with *Hibiscus syriacus* extract “D + HSE”. After the induction, period rats in the “C + HSE” and “D + HSE” groups were given *H. syriacus* extract (100 mg/kg/day) for four weeks by means of the gavage application, which means the administration of *H. syriacus* extract to the rats, through a tube leading down the throat to the stomach. The oral gavage method was applied efficiently and carefully, ensuring that the tested extract reached the stomach of the animal directly and in a measured way. This was important for the accuracy and consistency of the dose used in the study. During the four-week experimental period, daily food and liquid intake was monitored, and body weight was measured weekly across all experimental groups.

### 2.6. Preparation of Samples and Biochemical Testing

At the conclusion of the experimental period, the animals were fasted for 12 h and euthanized via cardiac puncture under anesthesia. Blood samples were collected in tubes designed for serum and plasma analyses and centrifuged at 1500 rpm for 10 min to separate serum and plasma. Heart, kidney, liver, and skeletal muscle tissues were removed immediately after blood collection and stored at −20 °C until they were washed with saline and studied.

Blood glucose levels (mg/dL) were measured weekly using a drop of blood from the tail of the rat and a glucometer (Optima, Taiwan, China). The levels of insulin, superoxide dismutase (SOD), and glutathione peroxidase (GSH-Px) were measured in the plasma, heart, skeletal muscle, liver, and kidney using ELISA kits from YL Biont (Shanghai, China). The measurements were performed according to the manufacturer’s protocol using a standard set of reagents from commercially available rat diagnostic kits.

For the lipid peroxidation test, blood was collected into tubes containing EDTA, and the plasma was separated and analyzed. Malondialdehyde (MDA) content in the plasma and tissues (heart, skeletal muscle, liver, and kidney) was measured using a spectrophotometric method [18]. The concentration of lipid peroxides in the tissues was expressed as nmol of MDA per mg of tissue. Total cholesterol (TC), triglyceride (TG), alanine aminotransferase (ALT), and aspartate aminotransferase (AST) levels in the serum were measured using commercial test kits (Fujifilm FDC, Tokyo, Japan) with an autoanalyzer (Fuji Dri-Chem, Tokyo, Japan).

### 2.7. Assessment of Antioxidant Capacity

The radical-scavenging activity of Hibiscus syriacus leaves was assessed using 2,2′-azino-bis-(3-ethylbenzothiazoline-6-sulfonic acid) diammonium salt (ABTS), following a previously described method with slight modifications. The absorbance was read at 735 nm using a microplate reader (BioTek, Winooski, USA). The Trolox equivalent antioxidant capacity (TEAC) was calculated from a Trolox standard curve and expressed as mg of Trolox equivalents (TE) per gram of dry sample.

### 2.8. Molecular Docking Study

In this study, the molecular docking effects of gallic acid with superoxide dismutase were investigated. The PDB entry 3GTV was selected as the appropriate receptor for the docking process. Molecular docking is highly important for examining ligand–protein interactions [19]. Molecular docking procedure in this study was conducted using the AutoDock Vina program [20]. Auto-Dock Tools were prepared and used for graphical interfaces. Discovery Studio 3.5 software was used for images [21].

### 2.9. Statistical Analysis

All statistical analyses were conducted using SPSS 28.0 for Windows. The results are presented as mean ± standard error. Based on the normality test results, the non-parametric Kruskal–Wallis test was used for the analysis. Post hoc comparisons were performed using Tamhane’s T2 test to identify differences between the groups. The level of significance was set at *p* ≤ 0.05.

## 3. Results

### 3.1. Chemical Composition of Hibiscus syriacus

**Figure 2:**
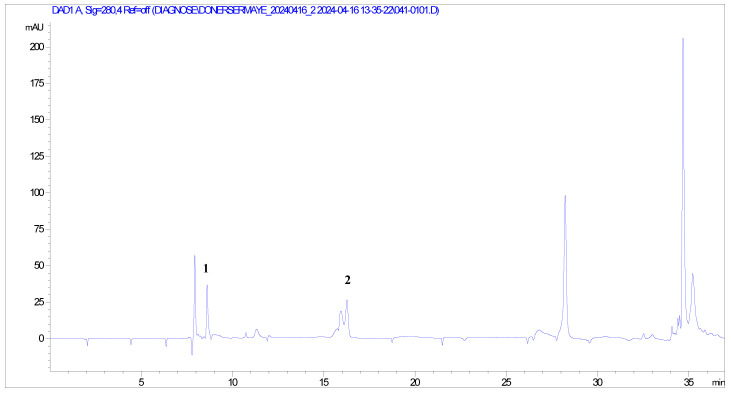
HPLC-DAD chromatograms of components in standards recorded at 360 nm. Quantification showed that *Hibiscus syriacus* contained. Peak 1: 7.22 ± 0.11 mg/L gallic acid (8.60 min), Peak 2: 12.71 ± 0.17 mg/L catechin (16.27 min).

The chemical constituents of *Hibiscus syriacus* were examined using HPLC-DAD analysis (Figure 2). Two peaks were observed with retention times of 8.60 and 16.27 min in the chromatogram, which were identified as gallic acid and catechin. Other peaks could not be identified because they were not present in the standard library. The contents of gallic acid as 7.22 ± 0.11 mg/L and catechin as 12.7 ± 0.17 mg/L in HSE were determined.

### 3.2. Impact of HSE on Body Weight, Food Intake, and Fluid Consumption in Normal and Diabetic Rats

**Figure 3:**
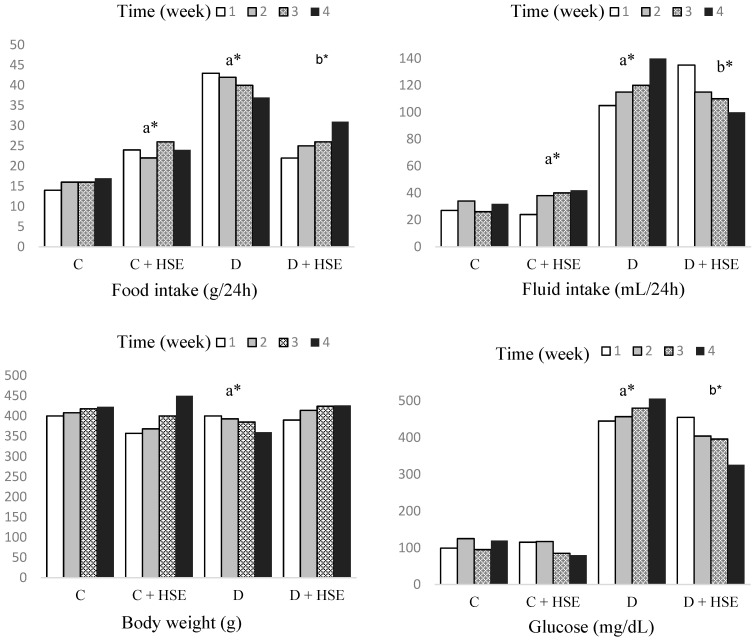
Food and fluid intake, body weight, and blood glucose changes over four weeks in both control and experimental groups of rats. a: Compared with control. b: Compared with diabetes group. Statistical significance: * *p* < 0.05, C: Control; C + HSE: Control + *Hibiscus syriacus* extract; D: Diabetes; D + HSE: Diabetes + *Hibiscus syriacus* extract.

In Figure 3, data illustrating changes in food and fluid intake, body weight, blood glucose levels, and insulin levels over a span of four weeks are presented for both the control and experimental groups of rats. The body weight loss–gain effect of HSE in normal and type 1 diabetic rats. Diabetic animals (D) exhibited a statistically significant reduction in body weight compared with the control group (C) (*p* < 0.05). As shown in Figure 2, daily oral treatment with HSE caused a weight-gaining effect on the treated rats, with the most pronounced and significant (*p* < 0.05) effect seen in the group treated with HSE (D + HSE) when compared with the diabetic group (D).

In the D group, food consumption was significantly increased compared with the C group (*p* < 0.05). In the C + HSE group, food consumption and fluid consumption were statistically higher than those in the C group (*p* < 0.05). Food and fluid intake were significantly decreased in the D + HSE group compared with the D group (*p* < 0.05).

### 3.3. Biochemical Analyses

**Figure 4:**
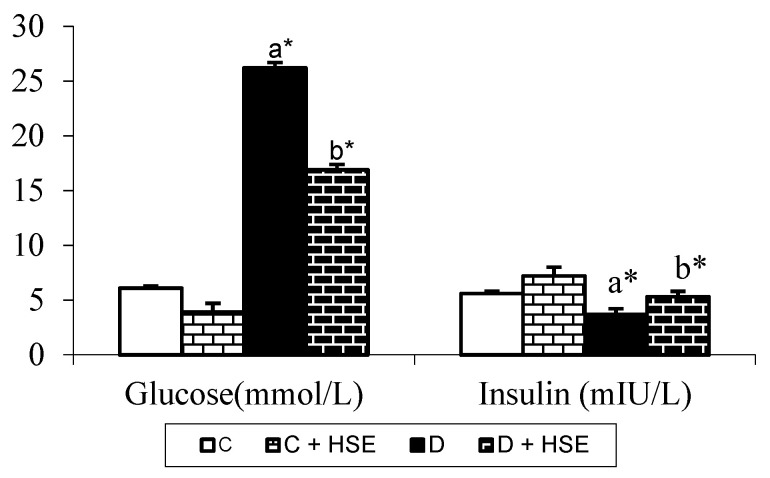
Blood glucose and plasma insulin values in the control and experimental groups of rats at the end of the four-week experimental period. a: Compared with control. b: Compared with diabetes group. Statistical significance: * *p* < 0.05. C: Control; C + HSE: Control + *Hibiscus syriacus* extract; D: Diabetes; D + HSE: Diabetes + *Hibiscus syriacus* extract.

Figure 4 depicts the effect of the daily oral administration of HSE on the blood glucose and plasma insulin levels.

In the D group, blood glucose levels were significantly increased, while insulin levels were significantly decreased compared to those in the C group (*p* < 0.05). Blood glucose was significantly decreased in the D + HSE group versus the D group; also, the serum insulin level was significantly increased the D + HSE group compared with the D group (*p* < 0.05).

**Figure 5:**
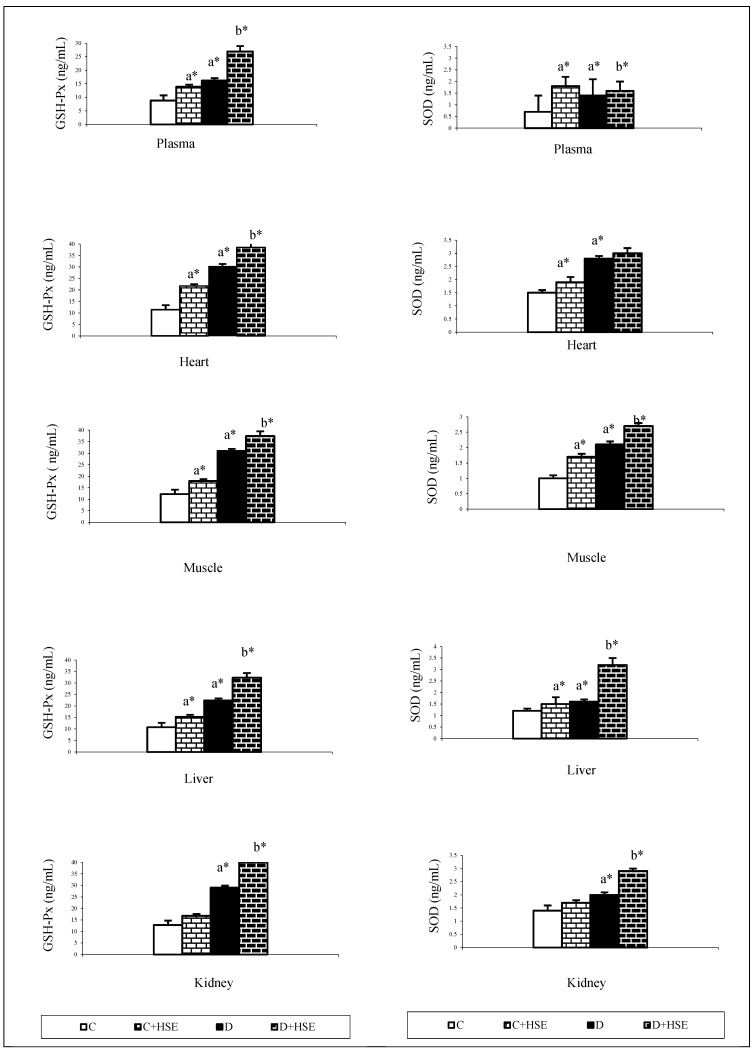
Glutathione peroxidase (GSH-PX) and superoxide dismutase (SOD) in control and experimental groups of rats. Values are expressed as mean ± SEM for rats each group (*n* = 9). a: Compared with control. b: Compared with diabetes group. Statistical significance: * *p* < 0.05. GSH-Px: Glutathione peroxidase; SOD: superoxide dismutase; C: Control; C + HSE: Control + *Hibiscus syriacus* extract; D: Diabetes; D + HSE: Diabetes + *Hibiscus syriacus* extract.

The SOD and GSH-Px levels in the plasma, heart, skeletal muscle, liver, and kidney tissues are shown in Figure 5. Both plasma and tissue (heart, muscle, liver, and kidney) GSH-Px and SOD activity were significantly higher in the diabetic group versus the control group (*p* < 0.05, 0.01). *H. syriacus* led to significant increases in these parameters in both the C + HSE and D + HSE groups compared to their respective C and D groups (*p* < 0.05, 0.01, Figure 5).

The MDA levels of plasma, heart, muscle, liver, and kidney tissues are shown in Table 1. The MDA levels of different tissues were significantly higher in group D than in the C group (*p* < 0.05, 0.01). However, *H. syriacus* significantly decreased plasma and different tissue MDA levels in the D + HSE group versus the D group (*p* < 0.05, 0.01, Table 1).

Serum levels of TC, TG, AST, and ALT are presented in Table 2. In the D group, TC, TG, AST, and ALT levels were significantly higher compared to the C group (*p* < 0.05, 0.01). In the D + HSE group, serum TC, TG, AST, and ALT levels showed a significant decrease compared to the D group (*p* < 0.05, 0.01).

### 3.4. Antioxidant Capacity

The antioxidant capacity of *Hibiscus syriacus* in vitro was assessed using the ABTS method, which revealed significant ABTS+ radical-scavenging activity. This activity was quantified using Trolox-equivalent antioxidant capacity (TEAC) values, which were recorded at 7.67 ± 0.09 mg. Trolox served as a positive control during the evaluation.

### 3.5. Molecular Docking

**Figure 6:**
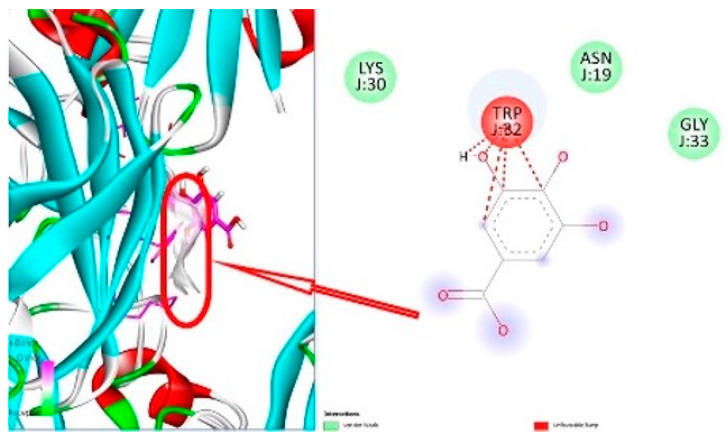
3D and 2D representation of molecular docking of gallic acid and superoxide dismutase enzyme (PDB ID 3GTV). 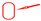: Interaction site.

Figure 6 illustrates that gallic acid, which has a binding affinity of −6.3 kcal/mol, could potentially alleviate oxidative stress, suggesting a decrease in cellular damage associated with diabetes.

## 4. Discussion

Streptozotocin (STZ), also known as N-methylnitrocarbamoyl-D-glucosamine, is a nitrosourea compound frequently used as a model to induce diabetes in rats and serves as a valuable tool for evaluating potential antidiabetic treatments. It is commonly employed in diabetes mellitus (DM) research because it has a specific affinity for pancreatic β-cells, resulting in a reduction in blood insulin levels and the development of hyperglycemia, effectively replicating the pathology of DM [22]. STZ’s mechanism involves the partial destruction of β-cells, which are responsible for producing insulin, thereby inducing diabetes in experimental animals [23]. This model, often induced by a combination of a high-energy diet and a low dose of STZ, is considered a useful approach for the preliminary assessment of diabetic effects, closely simulating the complex interplay between dietary factors and β-cell damage.

Despite the prevalence of pharmaceuticals for diabetes treatment, the use of medicinal plants for managing this condition has been shown to yield favorable outcomes. Herbal remedies and plant constituents, distinguished by minimal toxicity and the absence of adverse effects, have emerged as significant alternatives for diabetes management worldwide. Numerous studies have demonstrated the efficacy of medicinal plants with hypoglycemic properties in controlling diabetes. Herbal remedies, such as *Momordica charantia*, *Zingiber officinale*, *Gymnema sylvestre*, *Hoodia gordonii*, *Opuntia* spp., *Artemisia dracunculus* L., *Allium sativum*, *Ginkgo biloba*, *Panax* spp., *Aloe vera*, and *Hibiscus* spp., are among the valuable plants that have long been used as over-the-counter treatments for diabetes [24]. Flavonoids, tannins, phenolics, and alkaloids are the primary bioactive compounds utilized in diabetes management. The presence of these bioactive constituents highlights the therapeutic significance of these plants in combating diabetes [25].

The significance of dietary interventions in addressing hyperlipidemia and the associated oxidative stress cannot be overstated. It is not surprising that over the past decade, there has been a notable increase in the use of complementary and alternative medicines for managing various ailments, including diabetes mellitus and hyperlipidemia [26,27]. In addition to the reduced likelihood of side effects, many complementary, alternative, or traditional medicinal agents are derived from natural sources and are more cost effective [25].

There is growing interest in edible plants that contain antioxidants and health-promoting phytochemicals as potential therapeutic options [28]. These plants are often referred to as “medicinal plant foods”. One such example of these medicinal plant foods includes vegetables such as *Hibiscus syriacus*. This plant has been the subject of research and is recognized for its anti-inflammatory properties [29] as well as its pro-apoptotic, antibacterial, and antioxidant characteristics, which notably enhance blood circulation [30]. Nevertheless, the potential of *Hibiscus syriacus* L. as an antidiabetic agent has not been extensively investigated. In the current study, our objective is to explore the therapeutic capabilities of *Hibiscus syriacus* L. in diabetic rats induced by STZ and assess its impact on associated complications.

Body weight is an indicator of good health and efficient metabolic homeostasis [31]. From the results of this work, the differences in body weight between groups before the induction of diabetes were not significant (Figure 2). The significant decrease in body weight in diabetic rats is due to the deficiency of insulin; the fat and protein are catabolized (Figure 2). The improved body weight from the extracts of *H. syriacus* may be due to the alternative fats and tissue proteins that are broken down to produce energy that therefore compensate the loss in body weight and control of the hyperglycemic state in these rats and their energy intake. As seen in Figure 2, excessive water consumption in the diabetic group is due to high blood sugar. In diabetic animals, the body may also lose water as it tries to excrete excess glucose through urine. This situation can be explained by excessive thirst and, as a result, excessive water consumption. Many antidiabetic herbs have been reported with similar effects [32,33].

Glucose is a vital energy source for all cells and organs; however, high concentrations can lead to various complications. Therefore, regulating blood sugar levels is crucial for alleviating various problems associated with high glucose levels. Numerous studies have shown that various Hibiscus species can lower blood glucose levels and elevate serum insulin levels in rats with streptozotocin (STZ)- or alloxan-induced diabetes [33,34,35,36]. The oral administration of methanol extracts of *Hibiscus syriacus* led to a decrease in blood glucose and increase in insulin levels in STZ-induced diabetic rats (Figure 4).

Recent studies have identified organic acids (citric acid, fumaric acid, maleic acid), fatty acids (palmitic acid, oleic acid, linoleic acid, etc.) and various phenolic compounds from *Hibiscus syriacus* L., such as saponarin, coumaric acid, catechin, naringenin, kaempferol, quercetin, myricetin-galactoside, luteolin, and apigenin and its derivatives [14,37]. These components may work together or individually to stimulate insulin release, potentially helping to restore pancreatic β-cells or inhibiting the intestinal absorption of glucose [38]. The potential mechanisms underlying the hypoglycemic effect of *H. syriacus* may include several factors, such as the inhibition of glucose absorption in the intestines, suppression of the digestion of polysaccharides through the inhibition of amylase and glucosidase enzymes, and inhibition of hepatic gluconeogenesis. This plant extract may also stimulate glucose uptake in peripheral tissues. Although the effects may be immediate, the rise in glucose uptake and decrease in hepatic glucose production may also be facilitated by the stimulation of insulin secretion. Previous studies have demonstrated that *Hibiscus sabdarriffa* exhibits antidiabetic properties and leads to a significant decrease in serum glucose levels in rats [33]. Our results align with those observed in studies on Hibiscus sabdariffa [39,40]. It can be inferred that *H. syriacus* produces similar effects as Hibiscus sabdariffa by inhibiting the pancreatic α-amylase enzyme and slowing down the digestion of carbohydrates into more absorbable monosaccharides.

Gallic acid (GA) is a robust natural antioxidant that scavenges radicals, inhibits lipid peroxidation, chelates metal ions, and boosts the body’s defense system, reducing oxidative stress and enhancing pancreatic function and glucose regulation in diabetic rats [41]. GA activates antidiabetic properties not only through its antioxidant actions but also by stimulating peroxisome proliferator-activated receptor alpha (PPARγ), peroxisome proliferator-activated receptor gamma coactivator 1-alpha (PGC1α), and the Akt and 5′ adenosine monophosphate-activated protein kinase (AMPK) pathways, which enhance glucose uptake through glucose transport type 4 (GLUT4) and reduce mitochondrial dysfunction via uncoupling protein (UCP) protein regulation. The epicatechin and gallic acid mix significantly boosted β-cell function in diabetic models by increasing GLUT4 mRNA in fat cells, which supports better pancreatic insulin release and glucose management, including enhanced gluconeogenesis. The homeostasis model assessment (HOMA) was used to evaluate β-cell function and insulin sensitivity based on basal glucose and insulin levels, helping to elucidate the physiological interplay between glucose regulation and insulin dynamics effectively [42,43].

Catechins, a class of polyphenols, boost mitochondrial oxidative phosphorylation, resulting in increased ATP production. Furthermore, they promote mitochondrial biogenesis and diminish β-cell apoptosis through mechanisms involving mitochondria-associated enzymes, ultimately enhancing insulin secretion in response to glucose exposure [44,45].

Diabetes-related complications often arise from oxidative stress, a condition that develops when the production of oxidants exceeds their neutralization rate [46]. Antioxidant enzymes, including SOD, CAT, GPx, GSH, and GST, play a pivotal role in preserving the physiological levels of oxygen and hydrogen peroxide. They facilitate the conversion of oxygen radicals and help eliminate the organic peroxides produced upon exposure to STZ [47]. In this study, significant increases in antioxidant enzymes (SOD and GSH-Px) of all tissues were observed in diabetic rats compared to those in the control group (Figure 5). The elevated flow of glucose leads to an increase in oxidant generation and unsettles the equilibrium of antioxidant mechanisms through multiple interconnected routes [47]. Such an increase in enzymatic activity can be perceived as an intensified defense mechanism against the pronounced lipid peroxidation observed in diabetes. This increase in the functions of selected antioxidant enzymes might be attributed to an excessive compensatory response by HSE in response to the elevated synthesis of reactive oxygen species (ROS) during diabetes.

MDA, the end product of lipid peroxidation, serves as a sensitive indicator of oxidative stress, reflecting the extent of oxidative damage. In our study, we observed a significant decrease in malondialdehyde levels in the tissues of diabetic rats treated with Hibiscus extract (*p* < 0.05–0.01, Table 1). Furthermore, there was a discernible trend towards diminished tissue MDA levels in the C + HSE group (Table 1). Remarkably, the kidney MDA levels were significantly lower in the C + HSE group (Table 1, *p* < 0.05). As shown in Table 1, there was a statistically significant decline in D + HSE levels across all examined tissues (*p* < 0.05–0.01). In this study, we demonstrated the positive effect of HSE on MDA levels in vivo, and our results were consistent with previously published studies [48,49,50] and in publishing findings on *H. trionum* by Ziyanok [16].

The interplay between the liver and diabetes is both multifaceted and reciprocal. Research has indicated disturbances in lipid metabolism and glycogen-related conditions in diabetes, emphasizing the significance of evaluating liver function in diabetic individuals. Liver function tests include a range of biochemical analyses that measure various enzymes, proteins, and compounds produced by the liver [51,52]. Established markers of liver function, such as AST, ALT, and ALP, are disrupted in individuals with diabetes. In our study, we noted a significant reduction in the levels of AST and ALT, which are known to increase significantly under diabetic conditions, following treatment with Hibiscus (*p* < 0.05–0.01, see Table 2).

Diabetes mellitus is linked to notable alterations in plasma lipids and lipoproteins, increasing susceptibility to rapid atherosclerotic and cardiovascular complications. Numerous botanical extracts have shown potential in the management of blood sugar, lipid levels, and oxidative stress [48,53,54]. However, data on the lipid-lowering capabilities of *Hibiscus syriacus* under diabetic conditions are scarce. In our study, we identified a significant reduction in the plasma levels of total cholesterol (TC) and triglycerides (TG) in the D + HSE group compared to those in the D group (*p* < 0.05, Table 2). This reduction may be attributed to the polyphenolic compounds found in *H. syriacus*, which are known to influence the activity of enzymes pivotal to lipoprotein metabolism. *H. syriacus* L. is rich in various components, such as fatty acids, naphthalene pentacyclic triterpene esters and 9,9-O-Ferulolyl-(--)-sequisolaricinresinol, gamma-tocopherol, sitosterol, and squalene, all of which are known to be powerful antioxidants and antifungal properties [29]. Considering these findings, we conclude that absolute HSE could possibly inhibit the overproduction of ROS in all tissues.

**Figure 7:**
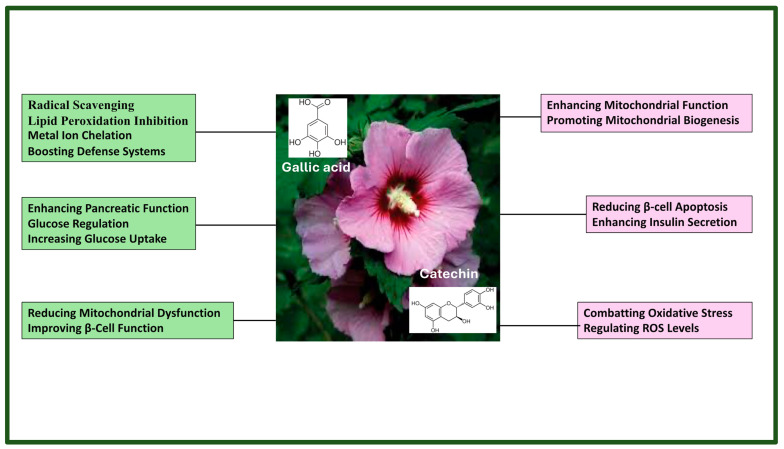
A scheme of the antidiabetic effects mechanisms of *Hibiscus syriacus*. Many studies have indicated that natural products can exhibit potent anti-inflammatory and antidiabetic effects at lower doses when used synergistically rather than individually. This is notable since natural products, such as fruits, vegetables, and endemic plants, contain diverse combinations of phytochemicals. These phytochemical mixtures significantly enhance their antidiabetic, antilipidemic, and anti-inflammatory properties as well as their therapeutic effects on various tissues [55,56,57,58].

The results of this study indicate that the therapeutic effects of *Hibiscus syriacus* on diabetes and its associated complications can be attributed to the synergistic effects of all the phytochemicals present in its content, enriched with beneficial flavonoids such as gallic acid, catechin, myricetin, apigenin, quercetin, kaempferol, etc. This highlights the significance of compound interactions within *Hibiscus syriacus* in mediating its antidiabetic and complication-mitigating effects. The mechanisms underlying the antidiabetic effects of *Hibiscus syriacus* are shown in Figure 7.

## 5. Conclusions

This study indicated that *Hibiscus syriacus* exerts its antidiabetic effects through multiple biochemical pathways. This plant mitigates oxidative stress by neutralizing free radicals and preventing lipid peroxidation, which helps regulate reactive oxygen species (ROS) levels. These benefits are due to the phytochemicals present in the plant, which enhance pancreatic function, improve glucose uptake, reduce mitochondrial dysfunction, boost β-cell function, and promote mitochondrial biogenesis. Collectively, these mechanisms contribute to a decrease in β-cell apoptosis and an increase in insulin secretion, thereby reinforcing the antidiabetic properties of the plantic properties.

Traditional medicine and ethnobotany provide a vast repository of information regarding the safety and biological effects of herbal products. Natural products (including herbal medicines) are the most valuable asset that nature has left us, with an indispensable role in the history of human health. Many herbs that are currently used to relieve hyperglycemia are rooted in traditional practices.

In conclusion, the multifaceted pharmacological activities of *Hibiscus syriacus* make it an effective natural complementary treatment for managing diabetes and related metabolic disorders. Our in silico analyses revealed strong molecular binding, which could contribute to a deeper understanding of the underlying mechanisms of diseases and facilitate the development of new therapeutic approaches. Further in vivo and molecular-level studies are essential to better understand the antidiabetic efficacy and bioavailability of *H. syriacus* extract. These studies are crucial for fully assessing the therapeutic potential of plants and laying the groundwork for future diabetes treatment strategies.

## Figures and Tables

**Table 1:** Malondialdehyde (MDA) control and experimental groups of rats. Values are expressed as mean ± SEM for rats each group (*n* = 9).

	C	C + HSE	D	D + HSE
Plasma MDA (nmol/mL)	2.2 ± 0.1	2.4 ± 0.5	7.0 ± 0.4 ^a^*	3.0 ± 0.1 ^b^*
Heart MDA (nmol/mg)	125.7 ± 1.4	105.4 ± 1.1	136.2 ± 1.3 ^a^*	111.1 ± 1.3 ^b^*
Muscle MDA (nmol/mg)	105.0 ± 1.1	96.3 ± 2.4	148.3 ± 2.4 ^a^**	128.6 ± 4.5 ^b^*
Liver MDA (nmol/mg)	124.0 ± 1.4	118.0 ± 3.1	168.5 ± 4.1 ^a^**	145.8 ± 3.6 ^b^**
Kidney MDA (nmol/mg)	134.4 ± 1.5	97.2 ± 1.7 ^a^*	174.9 ± 2.4 ^a^**	118.3 ± 3.1 ^b^**

^a^: Compared with control. ^b^: Compared with diabetes group. Statistical significance: * *p* < 0.05, ** *p* < 0.01. MDA: Malondialdehyde, C; Control C + HSE; Control + *Hibiscus syriacus* extract, D; Diabetes, D + HSE; Diabetes + *Hibiscus syriacus* extract.

**Table 2:** Triglyceride (TG), total cholesterol (TC), aspartate aminotransferase (AST), and alanine aminotransferase (ALT) levels in control and experimental groups of rats. Values are expressed as mean ± SEM for rats each group (*n* = 9).

	C	C + HSE	D	D + HSE
TG (mg/dL)	88.5 ± 0.5	67.3 ± 1.3	133.3 ± 1.4 ^a^*	99.4 ± 1.6 ^b^*
TC (mg/dL)	62.5 ± 1.5	55.0 ± 1.3	155.7 ± 3.3 ^a^*	103.6 ± 6.1 ^b^*
AST (U/L)	105.0 ± 1.1	101.0 ± 4.6	433.0 ± 3.4 ^a^**	179.7 ± 2.9 ^b^*
ALT (U/L)	64.0 ± 1.4	47.5 ± 5.2	194.0 ± 4.1 ^a^**	101.0 ± 2.0 ^b^**

^a^: Compared with control. ^b^: Compared with diabetes group. Statistical significance: * *p* < 0.05, ** *p* < 0.01. TG; Triglyceride, TC; Total Cholesterol, AST; Aspartate aminotransferase, ALT; Alanine aminotransferase, C; Control C + HSE; Control + *Hibiscus syriacus* extract, D; Diabetes, D + HSE; Diabetes + *Hibiscus syriacus* extract.

## Data Availability

The data presented in this study are available on request from the corresponding author.
