# Correction: Ziyanok-Demirtas, S. A Holistic In Silico and In Vivo Approach to Exploring the Antidiabetic, Antioxidant, and Hepatoprotective Properties of Rose of Sharon. *Life* 2024, *14*, 686

**DOI:** 10.3390/life15050788

**Published:** 2025-05-15

**Authors:** Sedef Ziyanok-Demirtas

**Affiliations:** Department of Biology, Faculty of Science and Arts, Bursa Uludag University, Bursa 16059, Turkey; sziyanok@uludag.edu.tr

## Error in Figure/Table

In the original publication [1], there was a mistake in Figures 3 and 5, as well as Table 1, when published. There was a typo in the unit. The corrected Figure 3 and Figure 5, as well as Table 1, appear below.

- In Figure 3, the glucose unit has been corrected to mg/dL.
- In Figure 5, only the liver and kidney Glutathione Peroxidase units have been corrected to ng/mL.
- In Table 1, the MDA units for heart, muscle, liver, and kidney should be nmol/mg.

## Error in Text

There was also an error in the Section 2.6, Preparation of Samples and Biochemical Testing, the second sentence of the second paragraph. The kit brand used was spelled incorrectly.

The original version stated the following:

The levels of insulin, superoxide dismutase (SOD), and glutathione peroxidase (GSH-Px) were measured in the plasma, heart, skeletal muscle, liver, and kidney using ELISA kits from Sunlong Biotech, Zhejiang, China.

The corrected version of the kits’ brand name stated as follows:

The levels of insulin, superoxide dismutase (SOD), and glutathione peroxidase (GSH-Px) were measured in the plasma, heart, skeletal muscle, liver, and kidney using ELISA kits from YL Biont (Shanghai, China).

The author states that the scientific conclusions are unaffected. This correction was approved by the Academic Editor. The original publication has also been updated.

## Figures and Tables

**Figure 3:**
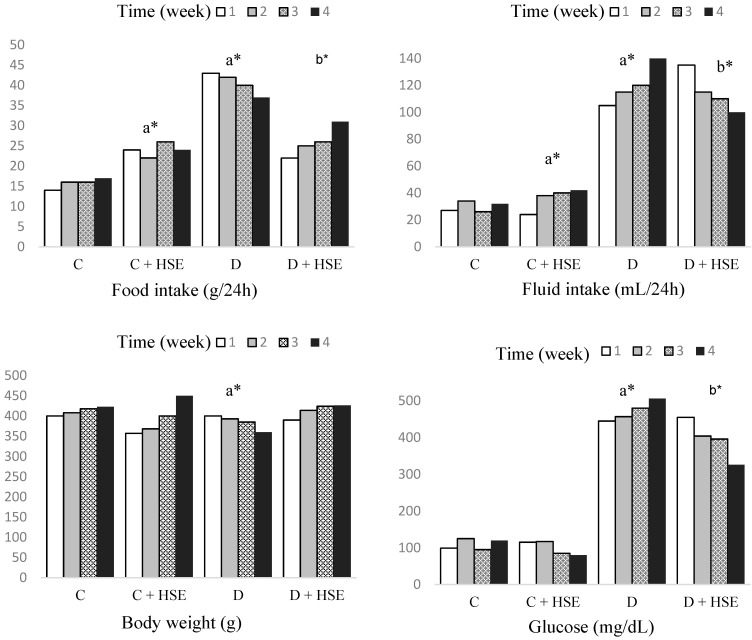
Food and fluid intake, body weight, and blood glucose changes over four weeks in both control and experimental groups of rats. a: Compared with control. b: Compared with diabetes group. Statistical significance: * *p* < 0.05, C: Control; C + HSE: Control + *Hibiscus syriacus* extract; D: Diabetes; D + HSE: Diabetes + *Hibiscus syriacus* extract.

**Figure 5:**
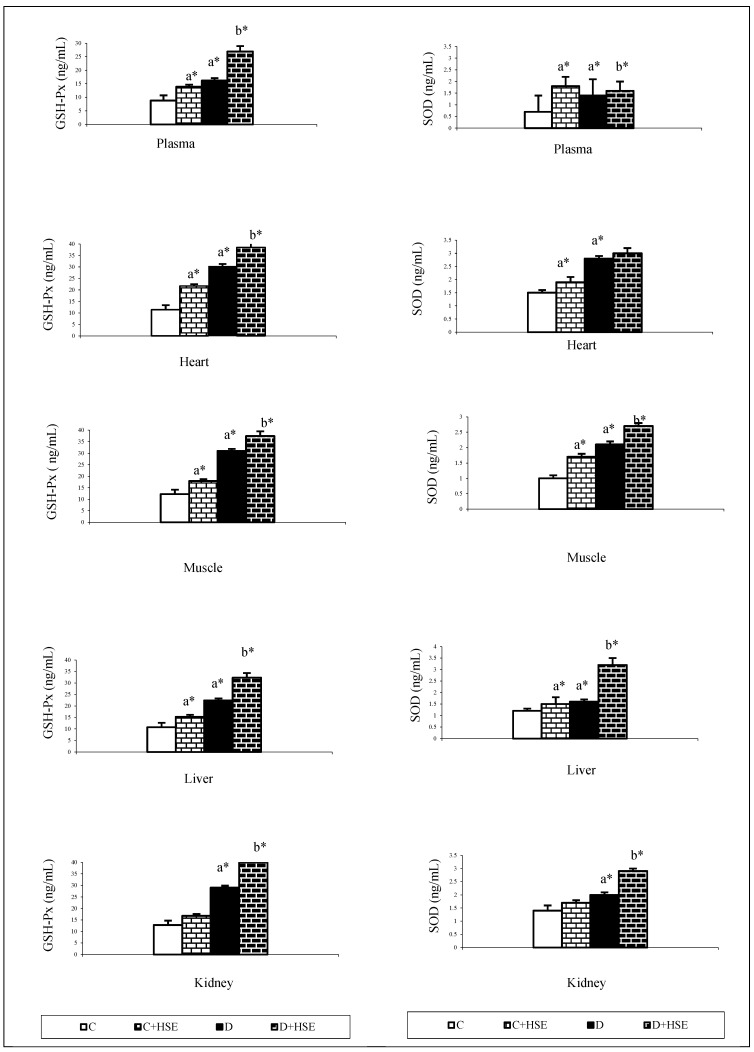
Glutathione peroxidase (GSH-PX) and superoxide dismutase (SOD) in control and experimental groups of rats. Values are expressed as mean ± SEM for rats each group (*n* = 9). a: Compared with control. b: Compared with diabetes group. Statistical significance: * *p* < 0.05. GSH-Px: Glutathione peroxidase; SOD: superoxide dismutase; C: Control; C + HSE: Control + *Hibiscus syriacus* extract; D: Diabetes; D + HSE: Diabetes + *Hibiscus syriacus* extract.

**Table 1:** Malondialdehyde (MDA) control and experimental groups of rats. Values are expressed as mean ± SEM for rats each group (*n* = 9).

	C	C + HSE	D	D + HSE
Plasma MDA (nmol/mL)	2.2 ± 0.1	2.4 ± 0.5	7.0 ± 0.4 ^a^*	3.0 ± 0.1 ^b^*
Heart MDA (nmol/mg)	125.7 ± 1.4	105.4 ± 1.1	136.2 ± 1.3 ^a^*	111.1 ± 1.3 ^b^*
Muscle MDA (nmol/mg)	105.0 ± 1.1	96.3 ± 2.4	148.3 ± 2.4 ^a^**	128.6 ± 4.5 ^b^*
Liver MDA (nmol/mg)	124.0 ± 1.4	118.0 ± 3.1	168.5 ± 4.1 ^a^**	145.8 ± 3.6 ^b^**
Kidney MDA (nmol/mg)	134.4 ± 1.5	97.2 ± 1.7 ^a^*	174.9 ± 2.4 ^a^**	118.3 ± 3.1 ^b^**

^a^: Compared with control. ^b^: Compared with diabetes group. Statistical significance: * *p* < 0.05, ** *p* < 0.01. MDA: Malondialdehyde, C; Control C + HSE; Control + *Hibiscus syriacus* extract, D; Diabetes, D + HSE; Diabetes + *Hibiscus syriacus* extract.
